# Cancer-specific survival after radical nephroureterectomy for upper urinary tract urothelial carcinoma: proposal and multi-institutional validation of a post-operative nomogram

**DOI:** 10.1038/bjc.2012.64

**Published:** 2012-02-28

**Authors:** D R Yates, V Hupertan, P Colin, A Ouzzane, A Descazeaud, J A Long, G Pignot, S Crouzet, F Rozet, Y Neuzillet, M Soulie, T Bodin, A Valeri, O Cussenot, M Rouprêt

**Affiliations:** 1 Academic Department of Urology of la Pitié-Salpêtrière Hospital, Assistance Publique-Hôpitaux de Paris, Faculté de Médecine Pierre et Marie Curie, University Paris VI, 47-83 Boulevard de l’Hopital, Paris 75013, France; 2 Academic Department of Urology and Statistics of Bichat-Claude Bernard Hospital, Assistance Publique-Hôpitaux de Paris, University Paris VII René Diderot, Paris, France; 3 Academic Department of Urology, CHRU Lille, University Lille Nord de France, Lille, France; 4 Academic Department of Urology, CHRU Limoges, University of Limoges, Limoges, France; 5 Academic Department of Urology, CHRU Grenoble, University of Grenoble, Grenoble, France; 6 Academic Department of Urology, Cochin Hospital, Paris, France; 7 Academic Department of Urology, Edouard Herriot Hospital, Claude Bernard Lyon 1 University, Lyon, France; 8 Department of Urology, Institut Mutualiste Montsouris, Paris, France; 9 Department of Urology, Foch Hospital, University of Paris-Ile de France Ouest, Suresnes, France; 10 Academic Department of Urology, CHRU Toulouse, University of Toulouse, Toulouse, France; 11 Academic Department of Urology, CHRU Poitiers, University of Poitiers, Poitiers, France; 12 Academic Department of Urology, CHRU Brest, University of Brest, Brest, France; 13 Academic Department of Urology of Tenon Hospital, Assistance Publique-Hôpitaux de Paris, Faculté de Médecine Pierre et Marie Curie, University Paris VI, Paris, France

**Keywords:** nomogram, urothelial carcinoma, renal pelvis: ureter, survival, prognosis

## Abstract

**Background:**

Owing to the scarcity of upper urinary tract urothelial carcinoma (UUT-UC) it is often necessary for investigators to pool data. A patient-specific survival nomogram based on such data is needed to predict cancer-specific survival (CSS) post nephroureterectomy (NU). Herein, we propose and validate a nomogram to predict CSS post NU.

**Patients and methods:**

Twenty-one French institutions contributed data on 1120 patients treated with NU for UUT-UC. A total of 667 had full data for nomogram development. Study population was divided into the nomogram development cohort (397) and external validation cohort (270). Cox proportional hazards regression models were used for univariate and multivariate analyses and to build a nomogram. A reduced model selection was performed using a backward step-down selection process, and Harrell's concordance index (c-index) was used for quantifying the nomogram accuracy. Internal validation was performed by bootstrapping and the reduced nomogram model was calibrated.

**Results:**

Of the 397 patients in the nomogram development cohort, 91 (22.9%) died during follow-up, of which 66 (72.5%) died as a consequence of UUT-UC. The actuarial CSS probability at 5 years was 0.76 (95% CI, 71.62-80.94). On multivariate analysis, T stage (*P*<0.0001), N status (*P*=0.014), grade (*P*=0.026), age (*P*=0.005) and location (*P*=0.022) were associated with CSS. The reduced nomogram model had an accuracy of 0.78. We propose a nomogram to predict 3 and 5-year CSS post NU for UUT-UC.

**Conclusion:**

We have devised and validated an accurate nomogram (78%), superior to any single clinical variable or current model, for predicting 5-year CSS post NU for UUT-UC.

Upper urinary tract urothelial carcinoma (UUT-UC) is a rare disease. Approximately as few as 3000 new cases per year occur in the United States and they account for only 5% of urothelial carcinomas (UC) overall (Hall *et al*, 1998; Edwards *et al*, 2006). Because of the scarcity of UUT-UC cases, clinical practice is guided by low levels of evidence and weak grades of recommendation (i.e., C) even in the most recent international guidelines (Roupret *et al*, 2011). Thus, it is often necessary for investigators to pool data when trying to draw meaningful conclusions from studies of UUT-UC. The majority of UUT-UCs are treated with radical nephroureterectomy (NU; plus bladder cuff removal) though there are few clinical situations nowadays in which one could try a conservative approach (Margulis *et al*, 2009; Ariane *et al*, 2012). Patients with UC infiltrating the upper urinary tract wall have a very poor prognosis. The survival rate at 5 years is <50% for those with stage T2–T3 tumours and less than <10% for those with T4 or N+/M+ tumours (Hall *et al*, 1998).

Nomograms are individualised prediction tools that can be used in either a pre- or post-treatment setting to predict an individual's probability of a particular endpoint, including disease stage, disease recurrence and cancer-specific survival (CSS; Chun *et al*, 2008). Nomograms have been widely publicised in prostate (D’Amico *et al*, 1999) and renal cancer (Kattan *et al*, 2001), but maybe their ultimate utility lies in rare clinical situations such as UUT-UC when subjective personal experience and strong clinical practice recommendations are lacking. Currently, there have been three nomogram models proposed for UUT-UC, two in a pre-operative setting to predict disease stage at the time of NU (Margulis *et al*, 2010; Favaretto *et al*, 2011) and a post-operative model to predict CSS (Jeldres *et al*, 2010). However, this latter nomogram is neither valid nor useable in a daily clinical environment as one of the four variables used in the prediction tool is an obsolete historical tumour grading system (Malmstrom *et al*, 1987) that is not used or recommended for grading UC (Mostofi *et al*, 1973; Epstein *et al*, 1998). Therefore, our aim was to propose and externally validate a new nomogram to predict 5-year CSS post NU using a multi-institutional French national database of UUT-UC and the recommended WHO grading system for UC.

## Patients and methods

### Patient population

Twenty-one French institutions contributed, to a national collaborative database, 1120 patients treated with NU (with bladder cuff removal) for non-metastatic UC of the renal pelvis and/or ureter between 1995 and 2010. From this database, 667 patients had complete data on age, sex, tumour stage (T), nodal status (N), tumour grade, margin status, associated carcinoma-*in-situ* and tumour location (renal pelvis or ureter). Clinical and pathological data were collected via medical and radiological file review at each centre. Descriptive statistics are displayed in Table 1 for both cohorts. All NU specimens were examined by dedicated genitourinary pathologists and processed according to standardised procedures. Tumours were staged according to the 2002 TNM classification by the American Joint Committee on Cancer-UICC (Greene *et al*, 2002) and tumour grading was assessed according to both the recommended 1973 WHO system and the 1998/2004 ISUP/WHO consensus classification (Mostofi *et al*, 1973; Epstein *et al*, 1998). Nodal status was determined by pathological assessment of retrieved lymph nodes at time of NU. In tumours synchronously involving the renal pelvis and ureter, the location was defined according to the site with the highest stage and/or grade. Collection and analyses of data were performed following Institutional Review Board approval from Assistance Publique Hopitaux Paris. Cause of death determination was performed by the responsible clinician based on medical note review and the authorised death certificate. Peri-operative deaths occurring within 30 days of surgery were censored. Before formal analysis, the database was frozen and a final dataset generated.

### Statistical analysis

**Figure 1:**
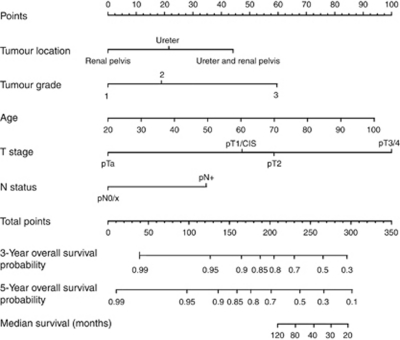
Proposed nomogram to predict 3- and 5-year CSS post NU for UUT-UC. To calculate survival probability; identify patient values on each axis then for each draw a vertical line upwards to the ‘points’ axis. This determines how many points each variable generates. Add the points for all variables and locate this sum on the ‘total points’ line. Then draw a vertical line downwards from this point and identify the 3- and 5-year probability of CSS. An online version of this risk calculator is available at http://pitie-salpetriere.aphp.fr/urologie (please click on ‘nomogram’).

**Figure 2:**
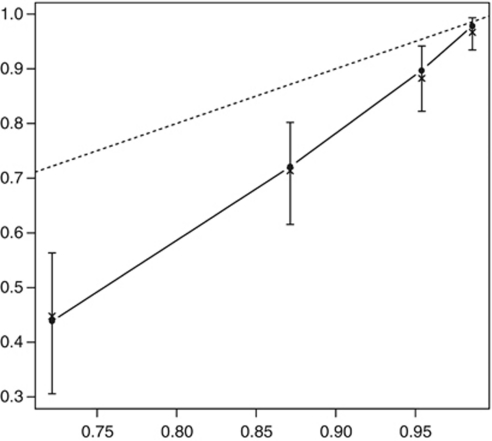
Calibration of the nomogram. The horizontal axis (*x*) is the nomogram-predicted probability of 5-year CSS post NU. The vertical axis (*y*) is actual 5-year CSS estimated with the Kaplan–Meier method. The continuous line in the middle is a reference line where an ideal nomogram would lie. The dotted line represents performance of the nomogram. Vertical bars represent 95% confidence intervals.

Actual survival was evaluated on censured data by the Kaplan–Meier (KM) method, and Cox proportional hazards regression models were used for univariate and multivariate analyses and to build a nomogram. The predictors analysed initially included age, sex, T stage, N stage, tumour grade, associated CIS, margin status and tumour location (renal pelvis or ureter). For the purpose of statistical analyses, the whole study population was divided into two cohorts. The nomogram development cohort and the external validation cohort constituted of 397 patients from 11 institutions (2/3 of the population) and 270 patients from 10 institutions (1/3 of the population), respectively. We initially developed several models. We chose a statistical method of training sets, which prevent over-learning and then we reused the entire data set to develop the final selected model using only variables that were significantly associated with CSS. A reduced model selection was then performed using a backward step-down selection process as described previously (Harrell *et al*, 1996). Harrell's concordance index (c-index) was used for quantifying the nomogram accuracy, and internal validation was performed on 200 samples by the bootstrapping technique (Bradley and Tibshirani, 1993; Harrell *et al*, 1996). For diseases with a low incidence, bootstrapping can improve the precision of the KM survival estimate, by providing a narrower CI. The final reduced model nomogram is displayed in Figure 1. Calibration plots (Figure 2) were generated to further validate the nomogram and this was assessed by grouping patients with respect to their nomogram-predicted probabilities and then comparing the mean of the actual observed KM estimate of 5-year CSS. Lastly, we used the external validation cohort to compare the final, reduced nomogram-predicted CSS versus the observed CSS at 5 years. All analyses were performed with R Version 2.13.1 (R Development Core Team, 2011) and Design package (Harrell, 2009). A *P*-value of <0.05 was considered significant.

## Results

Table 1 displays comparatively all the relevant information for both study cohorts. Specifically for the nomogram development cohort (*n*=397), the mean age was 68 (26–100). Tumour stages T1, T2, T3 and T4 occurred in 212 (53.4%), 36 (9%), 126 (31.7%) and 23 (5.9%), respectively. Tumour grades 1, 2 and 3 were seen in 30 (7.5%), 155 (39%) and 212 (53.5%), respectively. Of the 38.7% patients who underwent a systematic LND, 122 (30.7%) and 38 (9.6%) were staged as N0 and N1+, respectively. Associated CIS was identified in 16 (4%) patients. Tumours were located in the renal pelvis, ureter and synchronously in both in 229 (57.7%), 105 (26.4%) and 63 (15.9%) patients, respectively. Overall, NU with bladder cuff removal (NUC) was performed in 428 (64.2%) cases and an open approach was utilised in 498 (74.6%). A positive surgical margin was identified in 27 (6.8%) cases.

**Figure 3:**
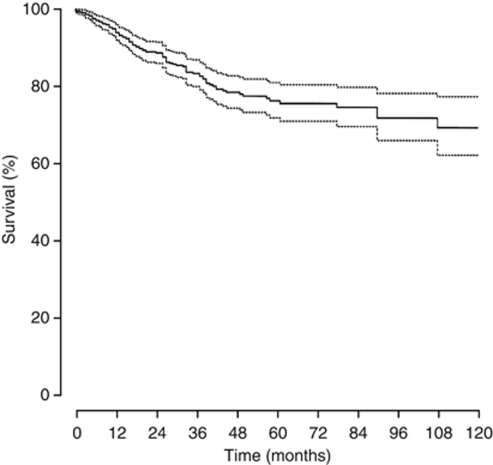
Kaplan–Meier plot of overall cancer-specific survival of the nomogram development cohort. The dotted curves represent the 95% confidence intervals. The continuous curve represents the cancer-specific survival.

**Figure 4:**
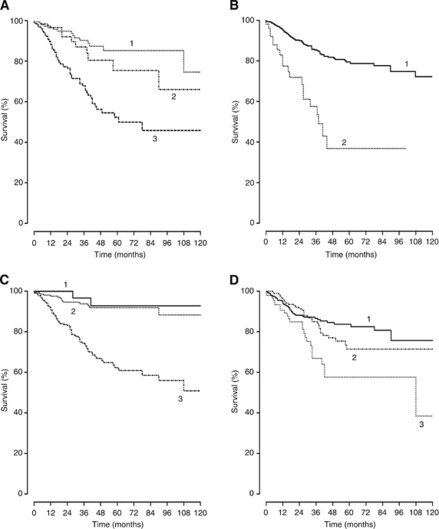
Kaplan–Meier plots of cancer-specific survival according to (**A**) T stage (1=T1; 2=T2; 3=T3/4), (**B**) N status (1=N+ 2=N0), (**C**) 1973 WHO tumour grade (1=grade 1; 2=grade 2; 3=grade 3) and (**D**) location (1=renal pelvis; 2=ureter; 3=both).

Of the 397 patients in the nomogram development cohort, 91 (22.9%) died during follow-up, of which 66 (72.5%) died as a consequence of UUT-UC. At 5 years after NU, 127 (20%) individuals remained at risk of death from UUT-UC. From KM analysis, the actuarial CSS probability at 5 years was 0.76 (95% CI, 71.62–80.94). KM plots of both overall 5-year CSS (Figure 3) and in respect of all nomogram included predictive variables are displayed in Figure 4A–D.

The results of univariate and multivariate Cox regression analysis models are highlighted in Table 2. In univariate analysis, age, T stage, N stage, tumour grade, age and location were all significant predictors of CSS. When applied to a multivariate model all variables except sex, margin status and associated CIS were significant. From this analysis, the predictive accuracy was calculated and the most important univariate predictor of CSS was T stage (*P*<0.0001). Cox regression coefficients were used to create a nomogram (Figure 1). As described, using a point scale from 0 to 100, each predictive variable is ‘weighted’ by assigning a point score. The point values for each variable are combined to give a total score, which is then correlated into the probability of 3- and 5-year CSS. Using all eight predictive variables in the full nomogram model achieved accuracy (c-index) of 0.75. As described, a backward step-down selection process was used to generate the most informative nomogram model that included age, T stage, N stage, tumour grade, age and location. This reduced nomogram model had an accuracy of 0.78. The nomogram is available as an online risk calculator at http://pitie-salpetriere.aphp.fr/urologie (please click on ‘nomogram’).

Bootstrapping of 200 samples was used to internally validate the reduced model nomogram and showed no deviation from the ideal. In the external validation cohort (*n*=270), the accuracy of the model was 0.78. Calibration plots of the nomogram-predicted probabilities and the actual number surviving in the external cohort are displayed in Figure 2.

## Discussion

Nomograms are now widely available for clinicians to utilise on an individual patient prediction basis and are superior to other prediction tools (Capitanio *et al*, 2008) or clinical judgement alone (Ross *et al*, 2002). They have been heavily publicised mostly in prostate (Kattan *et al*, 1998; D’Amico *et al*, 1999) and renal (Kattan *et al*, 2001; Karakiewicz *et al*, 2007) cancer but they also exist for bladder (Shariat *et al*, 2006) and penile cancer (Zini *et al*, 2009). The variety of the variables incorporated into nomograms has expanded from standard clinical and pathological data to include factors from modern imaging techniques (Favaretto *et al*, 2011) and biomarker studies (Shariat *et al*, 2008). They can be used in a pre- or post-treatment setting to predict an ever increasing number of surrogate endpoints including disease stage, biochemical recurrence, disease recurrence and survival specific to the cancer. However, it is undefined how popular and widely used are the current nomogram models, and it is possible to envisage that subjective clinical decision making based on experience and guideline evidence may still prevail for the common malignancies such as prostate and renal cancer. Because of the low incidence of UUT-UC, extensive experience on an individual clinician basis is lacking and it is in such clinical situations that nomograms may be ultimately beneficial. Currently, there are two models described in a pre-operative setting (Margulis *et al*, 2010; Favaretto *et al*, 2011). Used pre-operatively in UUT-UC, they may allow selection of patients who would benefit from neoadjuvant chemotherapy regimes, extended LND, better renal function at time of chemotherapy and possible avoidance of radical surgery for low-risk tumours. Post-operative predictions aid counselling and the rationalisation of adjuvant chemotherapy and the formal pathological variables are much better defined, validated and reproducible post-operatively.

Recently, Jeldres *et al* (2010) have proposed a post-operative model to predict survival post NU for UUT-UC. Using 17 Surveillance Epidemiology and Endpoint Results cancer registries, consisting of data from 1988 to 2006, they generated a database of 5918 patients. In the reduced model selection process generated from the nomogram development cohort, four variables (age, T stage, N stage and tumour grade) were found to be the most informative and parsimonious. The c-index after application of the nomogram to the external validation cohort was 75.4 *vs* 64.8% (*P*<0.001) for the comparative UICC staging system. However, the tumour grading system they utilised is historical and obsolete (Malmstrom *et al*, 1987) and not recommended by any international guidelines on the grading of UC (Roupret *et al*, 2011). Thus, this currently available nomogram for CSS post NU is of no use in daily current practice for clinicians that are likely to expect a more useful tool to predict survival. The 1973 WHO grading system is still widely used in most studies and in a clinical setting alongside the 1998/2004 ISUP/WHO recommendation (Mostofi *et al*, 1973; Epstein *et al*, 1998). We feel this invalidates this nomogram model as it is not applicable on a contemporary international setting.

Herein, we have used a now standardised statistical technique for nomogram development (Harrell *et al*, 1996; Iasonos *et al*, 2008) and externally validated it by dividing the study population into a nomogram development cohort and an external validation cohort. This is line with previously published nomogram models (Karakiewicz *et al*, 2007; Jeldres *et al*, 2010). Using a backward step-down selection process to select the most informative variables (age, T stage, N stage, tumour grade, age and tumour location) and multivariable Cox regression coefficient analysis, we were able to design a predictive nomogram (Harrell *et al*, 1996). Validation consisted of calibration (Figure 2), internal validation using Bootstrapping technique (Bradley and Tibshirani, 1993) and application of the reduced nomogram model to the external validation cohort. The accuracy of this nomogram was 78% and outperformed any other variable on univariate analysis (Table 2). This level of accuracy is universally in line with well-known published online models for prostate and renal cancer (Kattan *et al*, 1998, 2001; D’Amico *et al*, 1999; Karakiewicz *et al*, 2007).

We would like to address some limitations of our study, factors common to most published nomogram development series. The multi-institutional retrospective nature of the study creates variety in surgical technique and pathological review, but when it is necessary to maximise the statistical power of a study it is often required to pool data especially when the incidence of UUT-UC is low (3000 new cases per year in US compared to 53 000 for bladder UC; Edwards *et al*, 2006). The lack of central pathological review is an issue but the universal use of the 1973 WHO grading system is a positive not shared by other nomogram studies in UUT-UC (Jeldres *et al*, 2010). Thirty-eight percent (38.7%) of the study population had a formal LND, which is low. However, this is a reflection of the lack of standardisation, lack of templates and lack of knowledge of ‘landing sites’ for UC in the UUT. This percentage of LND is comparable to published series from high volume centres (Capitanio *et al*, 2009). Secondly, overall 36.6% did not have a bladder cuff removal, which increases the risk of recurrence and the influence that it can have on survival. When analysing patients with ureteric (unifocal or synchronous) tumours, the obligatory population for bladder cuff removal, this percentage rises to 90%. Again, similar figures exist for tertiary referral centres (Capitanio *et al*, 2009).

Combining clinico-pathological variables that are proven to be associated with clinical outcomes allows more accurate prediction than single-variable analysis. Nomograms provide the ideal format for such modelling and their graphical and online presentation make them user-friendly for both clinician and patient to aid a risk benefit discussion of available treatments. However, the overall accuracy of nomograms in all malignancies does not commonly exceed 80%. One strategy to improve this accuracy in UUT-UC is to collect data prospectively, more specifically, systematically and cleanly. However, such a scenario would be akin to utopia for UUT-UC and because the accuracy nomograms might depend on factors related to catchment areas, another strategy to improved prediction is to assemble larger datasets within the scientific community. But, one of the major drawbacks of a nomogram model is that it must be validated in external groups of patients with characteristics different from the original dataset before it can be generalised. Thus, we feel it will be appropriate in time for another group to validate our model in their population as we have done previously in cross cultural validation of popular renal and prostate nomograms (Hupertan *et al*, 2006; Roupret *et al*, 2009).

## Conclusion

Nomograms are established in modern medical practice and can be important tools to aid clinical decision making especially in situations were subjective clinical judgement may be difficult because of the scarcity of cases. We have devised and validated an accurate nomogram (78%), superior to any single clinical variable or current model, for predicting CSS post NU for UUT-UC.

## Figures and Tables

**Table 1:** Descriptive statistics of development cohort and external validation cohort

	**Nomogram development cohort**		**External validation cohort**	
**Variable**	**No. of patients**	**%**	**No. of patients**	**%**
*Centre*				
Lille University Hospital, Lille, France	84	21.1	NA	
Henri Mondor University Hospital, Paris, France	17	4.3	NA	
Cochin Hospital, Paris, France	122	30.6	NA	
Pitie-Salpetriere University Hospital, Paris, France	38	9.5	NA	
Edouard Herriot Hospital, Lyon, France	21	5.3	NA	
Reims University Hospital, Reims, France	14	3.5	NA	
Caen University Hospital, Caen, France	16	4	NA	
Val de Grace Military Hospital, Paris, France	30	7.5	NA	
Marseille University Hospital, Marseille, France	11	2.8	NA	
Toulouse Hospital, Toulouse, France	32	8	NA	
Nimes Hospital, Nimes, France	13	3.4	NA	
Brest Hospital, Amiens, France	NA		26	9.6
Lyon South Hospital, Lyon, France	NA		80	29.6
Rouen Hospital, Rouen, France	NA		44	16.3
Angers University Hospital, Angers, France	NA		19	7
Dijon University Hospital, Dijon, France	NA		8	3
Tenon Hospital, Paris, France	NA		28	10.4
Tours University Hospital, Tours, France	NA		6	2.2
La Conception Hospital, Marseilles, France	NA		11	4
Foch Hospital, Suresnes, France	NA		27	10
Clermont Ferrand University Hospital, Clermont Ferrand, France	NA		21	7.9
Total	397	100	270	100
				
*Age, years*				
Mean	68		69	
Range	26–100		34–94	
				
*Sex*				
Male	255	64.2	192	71.1
Female	142	35.8	78	28.9
				
*Tumour location*				
Renal pelvis	229	57.7	143	53
Ureteral	105	26.4	95	35.2
Both synchronously	63	15.9	32	11.8
				
*Bladder cuff removal*	258	65	165	61.1
				
*Lymph node dissection*				
Yes	160	40.3	97	35.9
				
*Pathological T category*				
pT1	212	53.4	127	47
pT2	36	9	31	11.5
pT3	126	31.7	94	34.8
pT4	23	5.9	18	6.7
				
*Pathological N category*				
pN0	122	30.7	73	27.1
pN1–3	38	9.6	24	8.9
PNx	237	59.7	173	64
				
*Tumour grade*				
I	30	7.5	22	8.5
II	155	39	175	64.8
III	212	53.5	73	26.7
				
Associated CIS	16	4	3	1.1
Positive surgical margin	27	6.8	34	12.6
Cancer-specific mortality	66	16.6	36	13.3
Overall mortality	91	22.9	56	20.7
				
*Follow-up, months*				
Mean	33.6		33.6	
Range	0–225		0–225	

Abbreviations: CIS=carcinoma *in situ*; NA=not applicable.

**Table 2:** Univariate and multivariate Cox regression analysis for both the full and reduced nomogram models

	**Univariate model**		**Full multivariate model**		**Reduced multivariate model**	
**Variables**	**HR**	***P*-value**	**HR**	***P*-value**	**HR**	***P*-value**
*T stage*		<0.0001		<0.0001		<0.0001
PT2 *vs* pT1	3.41	0.01	2.37	0.081	2.33	0.086
PT3 *vs* pT1	5.04	0.002	2.93	0.049	2.87	0.053
PT4 *vs* pT1	12.79	<0.0001	6.46	<0.0001	6.2	<0.0001
						
*N status*						
Positive *vs* negative	413	<0.0001	1.92	0.013	1.9	0.014
						
*Tumour grade*		<0.0001		0.039		0.026
2 *vs* 1	169	0.486	1.38	0.675	1.44	0.636
3 *vs* 1	8.35	0.003	2.88	0.158	3.08	0.13
						
*Tumour location*		0.002		0.007		0.005
Ureteral *vs* renal pelvis	1.23	0.378	2.22	0.002	2.62	0.001
Ureteral and renal pelvis *vs* renal pelvis	2.44	<0.0001	1.48	0.099	1.49	0.092
						
*Margin status*						
Positive *vs* negative	2.27	0.003	1.13	0.664		
						
*Associated CIS*						
Yes *vs* no	2.14	0.71	0.88	0.782		
						
Age	1.03	0.008	1.02	0.018	1.02	0.022
						
*Sex*						
Male *vs* female	0.96	0.835	1.24	0.31		
						
Predictive accuracy, %			75		78	

Abbreviations: CIS=carcinoma *in situ*; HR=hazard ratio.
